# Blood‐brain barrier water exchange measurements using FEXI: Impact of modeling paradigm and relaxation time effects

**DOI:** 10.1002/mrm.29616

**Published:** 2023-03-09

**Authors:** Elizabeth Powell, Yolanda Ohene, Marco Battiston, Ben R. Dickie, Laura M. Parkes, Geoff J. M. Parker

**Affiliations:** ^1^ Centre for Medical Image Computing, Department of Medical Physics and Biomedical Engineering University College London London UK; ^2^ Division of Psychology, Communication and Human Neuroscience, School of Health Sciences, Faculty of Biology, Medicine and Health University of Manchester Manchester UK; ^3^ Geoffrey Jefferson Brain Research Centre, Manchester Academic Health Science Centre University of Manchester Manchester UK; ^4^ Queen Square MS Centre UCL Institute of Neurology, University College London London UK; ^5^ Division of Informatics, Imaging and Data Sciences School of Health Sciences, Faculty of Biology, Medicine and Health, University of Manchester Manchester UK; ^6^ Bioxydyn Limited Manchester UK

**Keywords:** blood‐brain barrier, diffusion MRI, FEXI, permeability, water exchange

## Abstract

**Purpose:**

To evaluate potential modeling paradigms and the impact of relaxation time effects on human blood‐brain barrier (BBB) water exchange measurements using FEXI (BBB‐FEXI), and to quantify the accuracy, precision, and repeatability of BBB‐FEXI exchange rate estimates at 3 T.

**Methods:**

Three modeling paradigms were evaluated: (i) the apparent exchange rate (AXR) model; (ii) a two‐compartment model (2CM) explicitly representing intra‐ and extravascular signal components, and (iii) a two‐compartment model additionally accounting for finite compartmental $T_{1}$ and $T_{2}$ relaxation times ($2{\text{CM}}_{r}$). Each model had three free parameters. Simulations quantified biases introduced by the assumption of infinite relaxation times in the AXR and 2CM models, as well as the accuracy and precision of all three models. The scan–rescan repeatability of all paradigms was quantified for the first time in vivo in 10 healthy volunteers (age range 23–52 years; five female).

**Results:**

The assumption of infinite relaxation times yielded exchange rate errors in simulations up to 42%/14% in the AXR/2CM models, respectively. Accuracy was highest in the compartmental models; precision was best in the AXR model. Scan–rescan repeatability in vivo was good for all models, with negligible bias and repeatability coefficients in grey matter of ${\text{RC}}_{\text{AXR}}=0.43$ 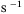, ${\text{RC}}_{2\text{CM}}=0.51$ 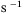, and ${\text{RC}}_{2{\text{CM}}_{r}}=0.61$ 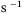.

**Conclusion:**

Compartmental modelling of BBB‐FEXI signals can provide accurate and repeatable measurements of BBB water exchange; however, relaxation time and partial volume effects may cause model‐dependent biases.

## Abstract 2

Click here for author‐reader discussions

## 1 INTRODUCTION

The blood‐brain barrier (BBB) separates the vasculature from brain tissue, and is important for maintaining normal brain function. Active transport of molecules necessary for metabolism is controlled by specialized proteins sited on the luminal and abluminal endothelial membranes, with passive diffusion restricted by tight junction proteins that seal together the endothelial cells. BBB dysfunction, where damage to the barrier allows pathogens and toxins to leak from the blood into the brain, is indicated in a majority of neurodegenerative diseases^1^, ^2^, ^3^, ^4^, ^5^, ^6^, ^7^, ^8^ as well as in stroke,^9^, ^10^ multiple sclerosis,^11^, ^12^, ^13^, ^14^ psychosis,^15^ brain tumors^16^, ^17^ and normal aging.^1^, ^2^, ^18^ There is increasing evidence to suggest that BBB alterations occur early in disease, so detecting subtle changes to BBB function may provide valuable insight into pathogenesis;^2^, ^4^ however, the primary established method for detecting elevated capillary leakiness—dynamic contrast‐enhanced MRI—has limited sensitivity to minor damage owing to the relatively large molecular size of the contrast agent chelate as well as signal confounds caused by a range of imaging artifacts.^5^, ^19^, ^20^, ^21^, ^22^

Measurements of water exchange across the BBB using MRI provide promising new biomarkers for identifying subtle changes in BBB function.^23^ Existing techniques for measuring water exchange fall broadly into three categories: (i) relaxometry‐based;^5^, ^8^, ^14^, ^24^ (ii) arterial spin labelling (ASL)‐based,^18^, ^25^, ^26^, ^27^, ^28^, ^29^, ^30^, ^31^, ^32^, ^33^, ^34^, ^35^ and; (iii) diffusion‐based.^36^, ^37^, ^38^ ASL‐based approaches currently dominate the available methods: contrast agents are not required, as is typical in relaxometry‐based approaches, and complimentary physiological parameters such as cerebral blood flow are also extracted. However, while altered exchange rates have successfully been detected in a range of diseases,^16^, ^27^, ^39^ ASL‐based approaches are often limited by low signal‐to‐noise ratio (SNR), resulting in long scan times. Diffusion‐based methods, which have only recently been proposed, have the potential to overcome some of these limitations.

Filtered‐exchange imaging (FEXI)^40^, ^41^, ^42^—a technique originally developed to measure water exchange across cell membranes by exploiting the difference in diffusivities between tissue compartments—can be adapted to measure exchange across the BBB (here denoted BBB‐FEXI).^36^, ^37^, ^38^ While initial BBB‐FEXI results show promise, current approaches rely on several critical assumptions and simplifications. For example, when applying FEXI to study cell membrane water exchange, biases due to intercompartmental $T_{1}$ and $T_{2}$ relaxation differences have been observed.^41^, ^43^ Relaxation time effects are inherently intertwined with exchange effects, as different rates of signal recovery and decay in different compartments will affect the observed relative signal fractions in a similar manner to exchange between compartments. Bias in BBB water exchange rate estimation is therefore to be expected if relaxation is not explicitly accounted for. While the impact of $T_{1}$ may be approximated and corrected,^41^ $T_{2}$ effects are harder to compensate for.^43^ This becomes of increasing importance for BBB‐FEXI measurements in the presence of pathology, where tissue $T_{1}$ and $T_{2}$ often change, frequently in tandem with BBB disruption.

A second potentially significant limitation of current BBB‐FEXI approaches is that compartmentalization has not been explicitly modeled. Instead, an apparent exchange rate (AXR) has been used to approximate the true water exchange rate,^36^ as was introduced for cell membrane measurements using FEXI.^41^, ^42^ AXR has the potential to be biased relative to the true underlying exchange rate owing to relaxation time differences^41^, ^43^ and does not provide insight into other potentially useful biomarkers, such as blood and tissue volume fractions and diffusivities. Finally, the accuracy, precision, and repeatability of BBB‐FEXI (for any modeling paradigm) has not yet been demonstrated.

The above considerations motivate the aims of this work, which are: (i) to evaluate compartmental modeling as a means of providing greater biophysical insight into BBB function; (ii) to quantify the impact of relaxation time effects on exchange rate estimation in both the compartmental and AXR models of BBB‐FEXI; (iii) to evaluate the accuracy and precision of the different modeling paradigms by employing signal simulations, and (iv) to evaluate for the first time the scan–rescan repeatability of BBB‐FEXI measurements in healthy subjects.

## 2 THEORY

### 2.1 Two‐compartment exchange model

**FIGURE 1:**
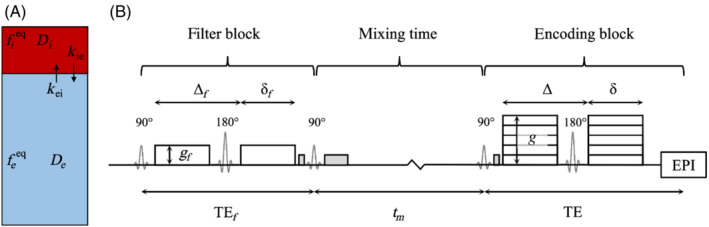
Blood‐brain barrier (BBB) water exchange measurements using filtered‐exchange imaging (BBB‐FEXI) signal model and pulse sequence diagram. (A). The two compartment model, composed of intra‐ and extravascular tissue components subscripted i and e, respectively. Each compartment has an associated equilibrium signal fraction ($f_{i}^{\text{eq}},f_{e}^{\text{eq}}$) and diffusivity ($D_{i}$, $D_{e}$) (note this is a pseudo‐diffusivity in the intravascular compartment). (B). Pulse sequence diagram. The diffusion filter block (subscripted f) and encoding block are defined by the gradient strength ($g_{f}$, g), duration ($\delta_{f}$, δ), and separation ($\Delta_{f}$, Δ). Dephasing gradients before and after the longitudinal magnetization storage pulses (second and third 90° pulses) and during the mixing time are shown in grey.

Given a two‐compartment system (here describing intra‐ and extravascular tissue components as in Figure 1A), the general solution for the magnetization at time t, M(t), given the magnetization state at time $t=t_{0}$, $M\left(t_{0}\right)$, and at equilibrium, $M^{\text{eq}}$, is:^44^, ^45^

(1) $$M\left(t\right)-M^{\text{eq}}=e^{-\left(q^{2}D+R^{\left(1,2\right)}+K\right)\left(t-t_{0}\right)}\left[M\left(t_{0}\right)-M^{\text{eq}}\right],$$

where

(2) $$\begin{array}{cc} M & =\left[\begin{array}{c} m_{i}\\ m_{e} \end{array}\right],\;D=\left[\begin{array}{cc} D_{i} & 0\\ 0 & D_{e} \end{array}\right],\;R=\left[\begin{array}{cc} R_{i} & 0\\ 0 & R_{e} \end{array}\right],\;\text{and}\\ K & =\left[\begin{array}{cc} \;k_{ie} & -k_{ei}\\ -\;k_{ie} & \;k_{ei} \end{array}\right], \end{array}$$

and subscripts *i* and *e* indicate the intra‐ and extravascular compartments. For longitudinal magnetization, $M_{i,e}^{\text{eq}}=M_{i,e}^{0}$, 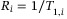 and 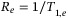; for transverse magnetization, $M_{i,e}^{\text{eq}}=0$, $R_{i}=1/T_{2,i}$ and $R_{e}=1/T_{2,e}$. The diffusion weighting is given by $b=q^{2}t={\left(\gamma \delta G\right)}^{2}t$, where q is the dephasing magnitude, γ is the gyromagnetic ratio, δ is the duration of the diffusion encoding gradients, and G is the diffusion encoding gradient strength. The extravascular diffusivity is represented by $D_{e}$ and the intravascular pseudo‐diffusivity by $D_{i}$. The intravascular‐to‐extravascular and extravasular‐to‐intravascular exchange rates are denoted $k_{ie}$ and $k_{ei}$, respectively. The exchange rate matrix K conserves the total magnetization such that $KM^{\mathrm{eq}}=0$ and $k_{ie}=k\;m_{e}^{\text{eq}}$.^44^

The diffusion filter in the FEXI pulse sequence (Figure 1B) applies a low *b*‐value to selectively suppress the signal from fast‐diffusing intravascular spins. Immediately after the diffusion filter, at time $t={\text{TE}}_{f}$ after the first 90° pulse, the magnetization is:

(3) $$M\left({\text{TE}}_{f}\right)=M^{0}e^{-\left(q_{f}^{2}D+R_{2}\right){\text{TE}}_{f}},$$

where $q_{f}$ is the dephasing magnitude of the filter gradients. During the mixing time $t_{m}$, in which the magnetization has been longitudinally stored by the second 90° pulse, exchange and $T_{1}$ relaxation govern its evolution such that at time $t={\text{TE}}_{f}+t_{m}$ the magnetization is:

(4) $$M\left({\text{TE}}_{f}+t_{m}\right)=M^{\text{eq}}+\left[M\left({\text{TE}}_{f}\right)-M^{\text{eq}}\right]e^{-\left(R_{1}+K\right)t_{m}}.$$

Dephasing gradients before the second and after the third 90° pulses select only the coherent magnetization encoded during the filter block and remove all other echo pathways, including any inflow effects (i.e. $M^{\text{eq}}=0$ in Equation 4). The eigenvalues and eigenvectors used in the determination of the matrix exponential in Equation (4) are provided in Appendix A.

The detected signal, that is the magnetization after the second diffusion encoding block at time $t={\text{TE}}_{f}+t_{m}+\text{TE}$, is:

(5) $$M\left({\text{TE}}_{f}+t_{m}+\text{TE}\right){|}_{q>0}=M\left({\text{TE}}_{f}+t_{m}+\text{TE}\right){|}_{q=0}\;e^{-q^{2}D\cdot \text{TE}},$$

where $M\left({\text{TE}}_{f}+t_{m}+\text{TE}\right){|}_{q=0}=M\left({\text{TE}}_{f}+t_{m}\right)e^{-R_{2}\cdot \text{TE}}$ is the magnetization at time $t={\text{TE}}_{f}+t_{m}+\text{TE}$ with q=0.

Simplifications to Equation (5) can be made in the absence of relaxation effects. As total magnetization is now preserved, the magnetization components can be described in terms of signal fractions, where at all times $f_{i}+f_{e}=1$. The signal after the filter block can then be described according to:

(6) $$S\left(b_{f}\right)=S_{0}\left(\left(1-f_{i}^{\text{eq}}\right)e^{-b_{f}D_{e}}+f_{i}^{\text{eq}}e^{-b_{f}D_{i}}\right),$$

where $S_{0}$ is the signal with $b_{f}=0$. Immediately after the diffusion filter, the intra‐ and extravascular signal fractions are described by $f_{i}^{0}$ and $f_{e}^{0}$, respectively. Exchange during the mixing time leads to a recovery of the fractional populations toward their equilibrium values subject to the exchange rate $k=k_{ie}+k_{ei}$:^40^

(7) $$f_{i}\left(t_{m}\right)=f_{i}^{\text{eq}}-\left(f_{i}^{\text{eq}}-f_{i}^{0}\right)e^{-kt_{m}}.$$

The signal measured after the encoding block in the absence of relaxation effects is then:^40^

(8) $$S\left(b_{f},t_{m},b\right)=S\left(b_{f},t_{m}\right)\left(\left(1-f_{i}\left(t_{m}\right)\right)e^{-bD_{e}}+f_{i}\left(t_{m}\right)e^{-bD_{i}}\right),$$

where b is the diffusion weighting of the encoding block and $S\left(b_{f},t_{m}\right)$ the filtered signal with b=0.

### 2.2 AXR model

The signal after a single diffusion experiment (i.e., Equation 6) can be approximated as:

(9) $$S\left(b\right)=S_{0}\;e^{-b\cdot \text{ADC}},$$

where the apparent diffusion coefficient is $\text{ADC}=f_{e}^{\text{eq}}D_{e}+f_{i}^{\text{eq}}D_{i}$. In an analogous manner, the FEXI signal can be approximated as:^41^

(10) $$S\left(b_{f},t_{m},b\right)=S\left(b_{f},t_{m}\right)e^{-b\cdot \text{ADC'}\left(t_{m}\right)},$$

where the mixing‐time‐dependent ADC, ${\text{ADC}}^{\prime }\left(t_{m}\right)$, is given by^41^

(11) $${\text{ADC}}^{\prime }\left(t_{m}\right)=\text{ADC}\left(1-\sigma e^{-t_{m}\cdot \text{AXR}}\right),$$

and the filter efficiency σ is defined as^41^

(12) $$\sigma =\frac{\left(D_{e}-D_{i}\right)\left(f_{e}^{\text{eq}}-f_{e}^{0}\right)}{\text{ADC}}.$$

In a two‐compartment system, the AXR is equivalent to the total exchange rate: $\text{AXR}=k_{ie}+k_{ei}$.

## 3 METHODS

Three modeling paradigms were evaluated: (i) the AXR model (Equations (10), (11), (12)), giving the apparent exchange rate denoted AXR; (ii) the two‐compartment model neglecting relaxation (Equations (6), (7), (8)), denoted 2CM and giving the average exchange rate denoted $k\left(=k_{ie}+k_{ei}\right)$, and; (iii) the two‐compartment model including relaxation (Equations(1), (2), (3), (4), (5)), denoted $2{\text{CM}}_{r}$ and giving the average exchange rate denoted $k_{r}$. The impact of relaxation time effects was first evaluated for the AXR and 2CM models using noise‐free simulations; the accuracy and precision of all three paradigms were then quantified under varying noise levels. Lastly, a repeatability study was conducted for all models in a cohort of healthy subjects.

All simulations and parameter estimations were performed in Matlab 2019b (The Mathworks). Sequence parameters for the simulation experiments were matched to the in vivo acquisitions (Table 1). Before fitting, signals were normalized using the signal at b=0 (in the encoding block) with corresponding filter *b*‐value and mixing time. Equilibrium blood signal fractions were fixed at 5% in grey matter (GM) and 3% in white matter (WM)^46^, ^47^, ^48^ for the compartmental models to stabilize fitting and to maintain the same number of free parameters as the AXR model. Free parameters in the AXR model were the ADC, AXR, and filter efficiency σ; for the compartmental models they were the intra‐ and extravascular diffusivities, $D_{i}$ and $D_{e}$, and exchange rate k (2CM) or $k_{r}$ ($2{\text{CM}}_{r}$). Parameters were constrained in all simulation experiments as follows: (i) $0.1{\text{ µm}}^{2}\text{/ms }\leq \text{ADC}\leq 3.5$ ${\text{ µm}}^{2}\text{/ms}$, 0≤σ≤1 and AXR>0 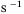 for the AXR model; (ii) $0.1{\text{ µm}}^{2}\text{/ms }\leq D_{e}\leq 3.5$ ${\text{ µm}}^{2}\text{/ms}$, $3{\text{ µm}}^{2}\text{/ms }\leq D_{i}\leq 30$ ${\text{ µm}}^{2}\text{/ms}$ and $k,k_{r}>0$ 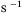 for the 2CM and $2{\text{CM}}_{r}$ models. A table of all model assumptions is provided in Table S1.

**TABLE 1:** Acquisition parameters.

	$T_{1}$w FFE	DW‐EPI	BBB‐FEXI			
Resolution (mm 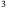 )	1×1×1	3×3×5	3×3×5			
Repetition time, TR (ms)	25	5000	5000			
Echo time, TE (ms)	3	62	62			
b‐values (${\text{ s/mm}}^{2}$)	—	0, 1000	0, 50, 100, 250, 1000			
Gradient directions	—	1, 6	3, 3, 3, 3, 3			
Averages	1	6	5			
Total volumes	1	42	300			
Scan time (min, s)	$4^{\prime }30^{\prime \prime }$	$4^{\prime }10^{\prime \prime }$	$25^{\prime }40^{\prime \prime }$			
Filter echo time, TE 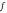 (ms)	—	—	38			
Filter *b*‐values, $b_{f}$ (${\text{s/mm}}^{2}$)	—	—	0	250	250	250
Mixing time, $t_{m}$ (ms)	—	—	20	20	200	400

*Notes*: All scans were acquired with SENSE acceleration factor 2. Total acquisition time was 36 min.

Abbreviations: BBB‐FEXI, blood‐brain barrier (BBB) water exchange measurements using filtered‐exchange imaging .

Relaxation times in vivo at 3 T were taken as: (i) $T_{1,i}=1.65$  s,^49^ $T_{2,i}=0.180$  s ^50^ in blood; (ii) $T_{1,e}=0.90$  s,^51^ 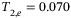  s ^51^ in WM; (iii) $T_{1,e}=1.50$  s,^51^ 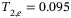  s ^51^ in GM.

### 3.1 Simulations

#### 3.1.1 Relaxation time effects

The effect of neglecting relaxation times during parameter estimation (equivalent to assuming infinite relaxation times) was investigated for a range of finite $T_{1}$ and $T_{2}$ values independently. Ground truth signals were simulated using the $2{\text{CM}}_{r}$ model for: (i) longitudinal relaxation times for both compartments between $0.7\text{ s}\leq T_{1,i},T_{1,e}\leq 2.5\text{ s}$ with $T_{2,i}=T_{2,e}=\infty$, and; (ii) transverse relaxation times between $0.05\text{ s}\leq T_{2,i},T_{2,e}\leq 0.20\text{ s}$ with $T_{1,i}=T_{1,e}=\infty$. For each experiment, 2500 parameter combinations were used. Other ground truth tissue parameters are provided in Table 2. The AXR and 2CM models (which assume infinite relaxation times) were fitted to the synthesised data and initialised using the ground truth parameters. The bias in AXR and k for each parameter combination was computed as the percent relative error between the ground truth ($k_{gt}$) and estimated ($k_{fit}$) exchange rate: $\text{error}=100\times \left(k_{fit}-k_{gt}\right)/k_{gt}$.

**TABLE 2:** Simulated parameter values.

	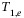 (s)	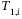 (s)	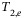 (s)	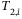 (s)	$f_{i}^{\text{eq}}$ (a.u.)	k ( 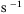 )
Relaxation time effects						
(i) Grey matter	0.7–2.5	0.7–2.5	∞	∞	0.05	3
(ii) Grey matter	∞	∞	0.05−0.20	0.05−0.20	0.05	3
(iii) Grey matter	0.7–2.5	1.65	∞	∞	0.05	3
(iv) Grey matter	∞	∞	0.05–0.20	0.18	0.05	3
Biases from fixed parameters						
(i) White matter	0.9	1.65	0.070	0.18	0.015–0.045	3
(i) Grey matter	1.5	1.65	0.095	0.18	0.025–0.075	3
(ii) White matter	0.77–1.04	1.65	0.070	0.18	0.03	3
(ii) Grey matter	1.28–1.73	1.65	0.095	0.18	0.05	3
(iii) White matter	0.9	1.65	0.06–0.08	0.18	0.03	3
(iii) Grey matter	1.5	1.65	0.08–0.11	0.18	0.05	3
Accuracy and precision						
(i) Grey matter	1.5	1.65	0.095	0.18	0.01–0.10	0.5–20

*Notes*: Ground truth generative parameter values are shown for each simulation experiment. In all cases the diffusivities of tissue and blood were $D_{e}=1{\text{ µm}}^{2}\text{/ms}$ and $D_{i}=10$ ${\text{ µm}}^{2}\text{/ms}$, respectively. All simulations used the $2{\text{CM}}_{r}$ model for signal generation.

The impact of mixing time on biases arising from intercompartmental $T_{1}$ differences was then investigated for three different maximum mixing times ($t_{m,\max}=300,400,500$  ms), and the impact of echo time on biases arising from intercompartmental $T_{2}$ differences was analysed for three combinations of filter and encoding echo times (${\text{TE}}_{f}/\text{TE}=20/40,38/62,60/80$  ms). All other simulation parameters are in Table 2.

#### 3.1.2 Biases from fixed parameters

Biases incurred by fixing $f_{i}^{\text{eq}}$ during parameter estimation were assessed for the 2CM and $2{\text{CM}}_{r}$ models; the impact of fixing relaxation times was additionally explored for the $2{\text{CM}}_{r}$ model.

Ground truth signals were generated using the $2{\text{CM}}_{r}$ model and a range of intravascular equilibrium signal fractions between $0.015<f_{i}^{\text{eq}}<0.045$ (WM) and $0.025<f_{i}^{\text{eq}}<0.075$ (GM). Parameter estimation was performed for each simulated signal using both the 2CM and $2{\text{CM}}_{r}$ models with the signal fraction fixed at $f_{i}^{\text{eq}}=0.03/0.05$ (WM/GM), thus assessing the effect of a ±50% error in fixed value. All other generative model parameters are provided in Table 2; $T_{1}$ and $T_{2}$ were assumed infinite for parameter estimation using the 2CM model and fixed to their ground truth values for the $2{\text{CM}}_{r}$ model.

Ground truth signals were then generated using the $2{\text{CM}}_{r}$ model and a range of extravascular longitudinal relaxation times between $0.77\text{ s}<T_{1,e}<1.04\text{ s}$ (WM) and $1.28\text{ s}<T_{1,e}<1.73\text{ s}$ (GM). Parameter estimation was performed fixing $T_{1,e}=0.90/1.50\text{ s}$ (WM/GM), reflecting an error of ±15% in fixed value. All other relaxation times were fixed to their ground truth values (Table 2). Finally, variability in ground truth transverse relaxation time was explored for 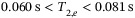 (WM) and 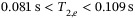 (GM) with values fixed at 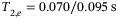 (WM/GM) during parameter estimation, again reflecting an error of ±15% in fixed values relative to the ground truth. Other relaxation times were again fixed to their ground truth values (Table 2).

All fitting was performed using a single initialisation at the ground truth parameter values.

#### 3.1.3 Accuracy and precision

The accuracy and precision of exchange rate estimates were evaluated under varying noise conditions for each modeling paradigm. Ground truth signals were generated using the $2{\text{CM}}_{r}$ model for 100 parameter combinations between $0.01\leq f_{i}^{\text{eq}}\leq 0.10$ and 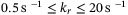. All other generative tissue parameters were invariant (Table 2). Gaussian noise was added to give 1000 noisy signals for each parameter set with SNR=60,100 (representative of the in vivo SNR) in the equilibrium signal (i.e., with $b_{f}=0$ ${\text{ s/mm}}^{2}$, $t_{m}=20$  ms, b=0 ${\text{ s/mm}}^{2}$). Fitting was performed as previously described, now using 20 initial values uniformly distributed between the respective parameter bounds; initial values for the exchange rate were distributed between the ground truth value ±50%. Accuracy was defined as the percent relative error of the median fitted value and precision as the interquartile range of fitted values. Extreme exchange rate estimates—defined as ≥40 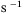
—were discarded from calculations.

### 3.2 MRI experiments

#### 3.2.1 Data acquisition

Ten healthy volunteers (age range 23–52 years; five female) were each scanned twice on a 3 T Philips Ingenia CX system (Philips Healthcare) using a 32‐channel head coil in accordance with local ethics guidelines. The second scan was conducted in the same session for nine of the volunteers (subjects repositioned between scans); for one volunteer their second scan was 6 weeks after the first. Whole brain diffusion‐weighted imaging (DWI) and $T_{1}$‐weighted images were collected for registration and segmentation purposes; an additional DWI with reversed phase‐encoding was acquired without diffusion weighting for susceptibility distortion correction. Single slice BBB‐FEXI data were acquired using a double diffusion encoding sequence developed in‐house. All acquisition parameters are provided in Table 1. Subsets of the full BBB‐FEXI acquisition were formed to create different protocols for the AXR and compartmental modeling paradigms. The AXR subset contained only data acquired with encoding b=0,250 ${\text{ s/mm}}^{2}$; all five repetitions of each acquisition were used, giving 120 volumes in total. The compartmental modeling subset contained two repetitions of the data acquired with all five encoding *b*‐values, again giving 120 volumes in total and matching the AXR dataset for total acquisition time (11 min).

#### 3.2.2 Data analysis

The DWI were corrected for susceptibility effects using FSL's *topup* tool.^52^, ^53^ The $T_{1}$‐weighted image was registered to the DWI with b=0 ${\text{ s/mm}}^{2}$,^54^ then segmented into WM, GM, and CSF using FSL FAST.^55^ The MNI template was also registered to the DWI and the deformation field used to propagate the Harvard‐Oxford atlas^56^ into the native space of each volunteer. Each BBB‐FEXI acquisition was then registered to the DWI using their respective b=0 ${\text{ s/mm}}^{2}$ volumes and corrected for susceptibility distortions using the previously estimated off‐resonance warp field. SNR in the BBB‐FEXI data was calculated using the mean and SD of the five repetitions with $b_{f}=0$ ${\text{ s/mm}}^{2}$, $t_{m}=20$  ms, b=0 ${\text{ s/mm}}^{2}$.

Exchange rate estimates were obtained for each modeling paradigm using the relevant data subsets. Voxel‐wise fitting was performed in Matlab 2019b using the Nelder‐Mead nonlinear minimization method. Parameters were constrained as follows: (i) $0.1{\text{ µm}}^{2}\text{/ms}\leq \text{ADC}\leq 3.5$ ${\text{ µm}}^{2}\text{/ms}$, 0.1≤σ≤1.0, 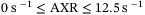 for the AXR model; (ii) $0.1{\text{ µm}}^{2}\text{/ms}\leq D_{e}\leq 3.5{\text{ µm}}^{2}\text{/ms}$, $3{\text{ µm}}^{2}\text{/ms}\leq D_{i}\leq 30$ ${\text{ µm}}^{2}\text{/ms}$, 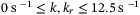 for the 2CM and $2{\text{CM}}_{r}$ models. Regional exchange rate maps were created using the median voxel‐wise estimate in each atlas ROI.

Bland–Altman plots were generated to assess exchange rate bias and variability between repeat scans. The repeatability coefficient was calculated for each modeling paradigm as: $\text{RC}=1.96\sqrt{2}\sigma_{w}$, with $\sigma_{w}$ the within‐subject variance.^57^ This quantified the smallest significant difference that may be observed between scan and rescan estimates at the 95% confidence level. Statistically significant differences (α=0.05) between exchange rate estimates from different modeling paradigms were calculated using a two‐sample *t*‐test on subject‐wise median WM/GM values; multiple comparisons were accounted for using the Bonferroni correction.

## 4 RESULTS

### 4.1 Simulations

#### 4.1.1 Relaxation time effects

**FIGURE 2:**
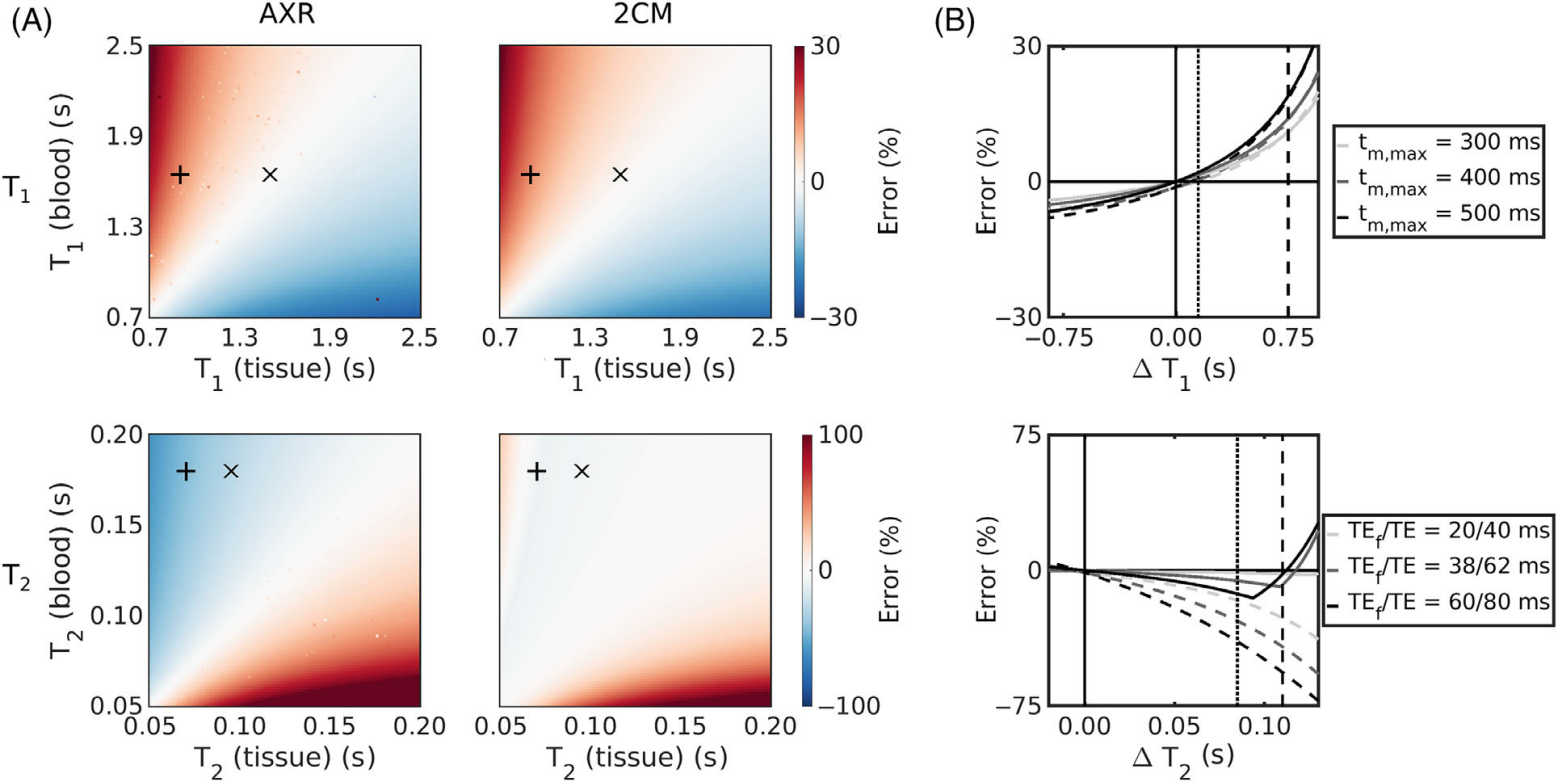
Assumption of infinite relaxation times. (A) The errors in exchange rate estimates are shown for a range of finite compartmental $T_{1}$ (top row) and $T_{2}$ (bottom row) values for the AXR (left column) and 2CM (right column) models. Expected blood/tissue values in white matter (WM) (+) and grey matter (GM) (×) are highlighted. (B) The impact of different maximum mixing times on the error in exchange rate estimates is shown for a range of $T_{1}$ differences (top); the impact of different echo times is shown for a range of $T_{2}$ differences (bottom). The AXR and 2CM models are represented by the dashed and solid lines, respectively. Expected relaxation time differences (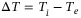) in WM and GM in vivo are indicated by the vertical dashed and dotted lines, respectively.

Figure 2A shows the bias in exchange rate estimates arising from finite compartmental relaxation times under the assumption of infinite relaxation times during model fitting. For typical blood and tissue $T_{1}$ values in vivo, errors using the AXR and 2CM models were similar at approximately 14%/1% in WM/GM, respectively. Errors from $T_{2}$ differences were high for the AXR model at 42%/28% (WM/GM); errors in the 2CM model were considerably lower at 8%/6% (WM/GM).

Reducing the echo times or maximum mixing times lowered the incurred biases (Figure 2B). For example, a reduction in echo times from ${\text{TE}}_{f}/\text{TE}=38\text{ ms}/62\text{ ms}$ to ${\text{TE}}_{f}/\text{TE}=20\text{ ms}/40\text{ ms}$ reduced the $T_{2}$ bias in AXR estimates by almost 40% to approximately 26%/17% (WM/GM).

#### 4.1.2 Biases from fixed parameters

**FIGURE 3:**
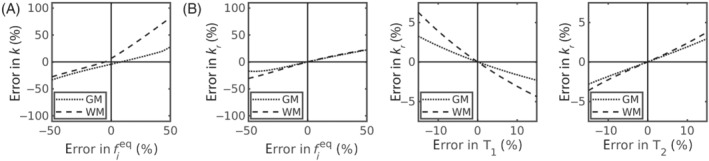
Biases in the compartmental models from fixed parameters. (A) Error in k from the 2CM model arising from errors in fixed $f_{i}^{\text{eq}}$ values. (B) Error in $k_{r}$ from the $2{\text{CM}}_{r}$ model arising from errors in fixed $f_{i}^{\text{eq}}$ (left), $T_{1,e}$ (center) and $T_{2,e}$ (right) values. Note that in both (A) and (B) the ±50% error in $f_{i}^{\text{eq}}$ covers the approximate range $0.015<f_{i}^{\text{eq}}<0.045$ for ground truth white matter values (where fixed $f_{i}^{\text{eq}}∼0.030$) and $0.025<f_{i}^{\text{eq}}<0.075$ for ground truth grey matter values (where fixed $f_{i}^{\text{eq}}∼0.050$). Note also the change in scale for errors arising from $T_{1}$ and $T_{2}$ versus $f_{i}^{\text{eq}}$.

Figure 3 quantifies the biases incurred by fixing parameters in the compartmental models during parameter estimation. Major biases were observed in the 2CM model, particularly in WM: an alteration of ±50% in underlying $f_{i}^{\text{eq}}$ (relative to the value fixed during fitting) incurred biases up to 82%/33% in WM/GM k estimates respectively (Figure 3A). However, note that a ±50% error in $f_{i}^{\text{eq}}$ covers the wide ranges of $0.015<f_{i}^{\text{eq}}<0.045$ for WM (where fixed $f_{i}^{\text{eq}}=0.030$) and $0.025<f_{i}^{\text{eq}}<0.075$ for GM (where fixed $f_{i}^{\text{eq}}=0.050$). Biases were not as severe for the $2{\text{CM}}_{r}$ model, with the same ±50% error in fixed $f_{i}^{\text{eq}}$ producing a 31/22% error in WM/GM $k_{r}$ estimates (Figure 3B).

Fixing relaxation times had minimal impact: an error of ±15% in fixed $T_{1,e}$ or $T_{2,e}$ relative to ground truth values induced biases in the estimated $k_{r}$ under 6% (Figure 3B).

#### 4.1.3 Accuracy and precision

**FIGURE 4:**
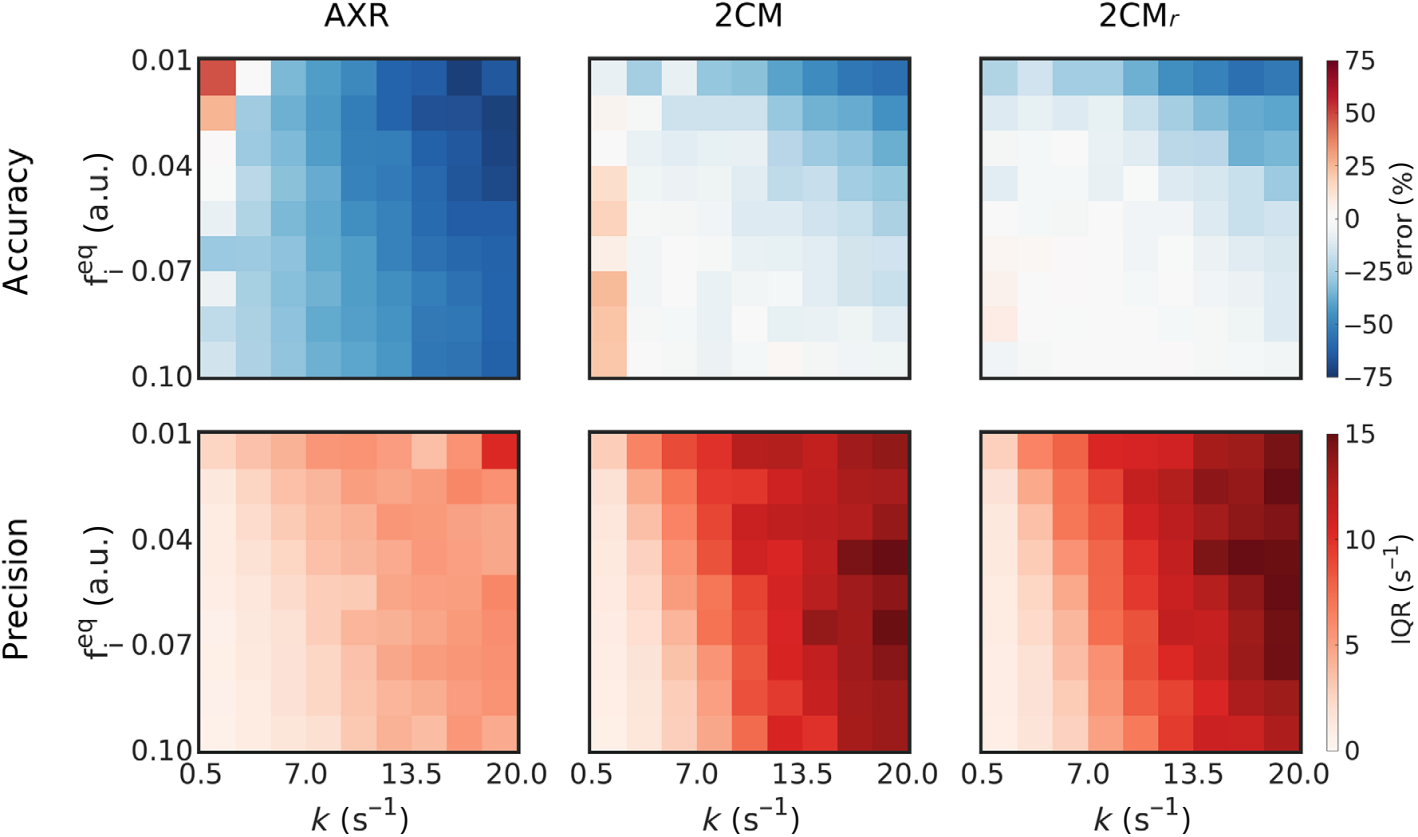
The accuracy (top row) and precision (bottom row) of estimated exchange rates are shown for a range of underlying blood volume fractions ($f_{i}^{\text{eq}}$) and exchange rates (k) for the AXR model (left column), 2CM model (centre column) and $2{\text{CM}}_{r}$ model (right column) at SNR=60.

Figure 4 shows the accuracy and precision in estimated exchange rates at SNR=60 as a function of underlying $f_{i}^{\text{eq}}$ and k for each modeling paradigm. The exchange rate was underestimated for the majority of tissue parameter combinations in all modeling paradigms; however, biases were greater in the AXR model than in either the 2CM or $2{\text{CM}}_{r}$ models. Accuracy was poorest for parameter combinations with low $f_{i}^{\text{eq}}$ and fast k in all modeling paradigms. Precision was also worse (interquartile range was greatest) for the low $f_{i}^{\text{eq}}$ and fast k parameter combinations, particularly for the 2CM and $2{\text{CM}}_{r}$ models.

**FIGURE 5:**
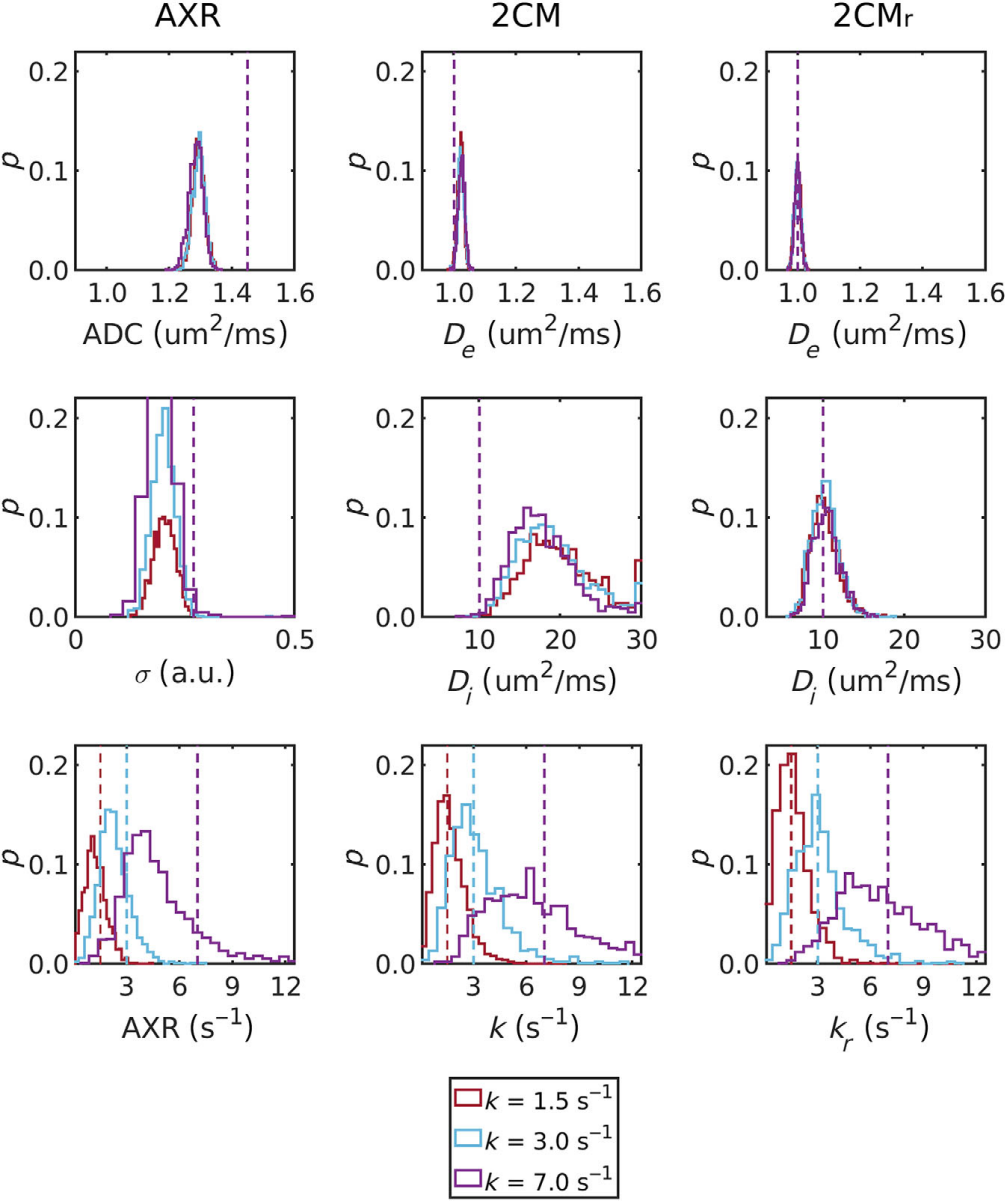
Model parameter probability distributions are shown for the AXR (left column), 2CM (center column) and $2{\text{CM}}_{r}$ (right column) models for three sets of generative parameter values ($f_{i}^{\text{eq}}=0.05$; k=1.5,3.0,7.0 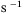; SNR=100). Ground truth (generative) values are represented by the dashed lines.

Figure 5 shows the distributions of all estimated parameters for three sets of tissue parameters ($f_{i}^{\text{eq}}=0.05$; k=1.5,3.0,7.0 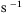) at SNR=100 (distributions at SNR=60 can be found in the Figure S3). Biases were evident in all parameters of the AXR model, with the AXR and ADC notably underestimated. Most striking in the 2CM model were the distributions of $D_{e}$ values, in which median values were approximately 85% greater than ground truth values. Minimal biases were observed in parameters of the $2{\text{CM}}_{r}$ model.

**TABLE 3:** Summary statistics.

		ADC (${\text{ µm}}^{2}\text{/ms}$)		σ (a.u.)		AXR ( 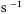 )	
		WM	GM	WM	GM	WM	GM
AXR	Mean (SD), scan 1	0.88 (0.04)	1.19 (0.08)	0.14 (0.02)	0.18 (0.03)	2.10 (0.39)	1.53 (0.47)
	Mean (SD), scan 2	0.89 (0.03)	1.19 (0.09)	0.15 (0.03)	0.18 (0.03)	1.92 (0.23)	1.35 (0.29)
	RC	0.004	0.021	0.002	0.002	0.29	0.43
	CoV (%)	4.21	7.39	19.5	16.2	16.3	29.2
		$D_{e}$ ( 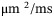 )		$D_{i}$ ( 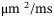 )		k ( 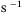 )	
		WM	GM	WM	GM	WM	GM
2CM	Mean (SD), scan 1	0.69 (0.03)	0.85 (0.04)	14.8 (1.0)	13.3 (1.5)	3.11 (0.43)	2.23 (0.46)
	Mean (SD), scan 2	0.69 (0.02)	0.85 (0.05)	15.1 (1.1)	13.6 (0.9)	2.86 (0.37)	2.16 (0.40)
	RC	0.002	0.006	3.14	4.32	0.44	0.51
	CoV (%)	3.60	5.50	7.18	9.31	13.4	20.4
		$D_{e}$ ( 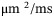 )		$D_{i}$ ( 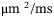 )		$k_{r}$ ( 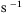 )	
		WM	GM	WM	GM	WM	GM
$2{\text{CM}}_{r}$	Mean (SD), scan 1	0.66 (0.03)	0.83 (0.04)	8.42 (0.69)	10.2 (1.0)	2.95 (0.27)	2.27 (0.49)
	Mean (SD), scan 2	0.66 (0.02)	0.83 (0.05)	8.43 (0.68)	10.7 (0.9)	2.90 (0.55)	2.18 (0.45)
	RC	0.002	0.006	1.29	2.55	0.52	0.61
	CoV (%)	3.71	5.58	8.06	9.14	14.7	22.7

*Notes*: The mean and SD of median voxel‐wise parameter values across subjects is shown for all modeling paradigms for scans 1 and 2, along with the repeatability coefficients (RC) and coefficients of variation (CoV).

Abbreviations: GM, grey matter; WM, white matter.

### 4.2 MRI experiments

**FIGURE 6:**
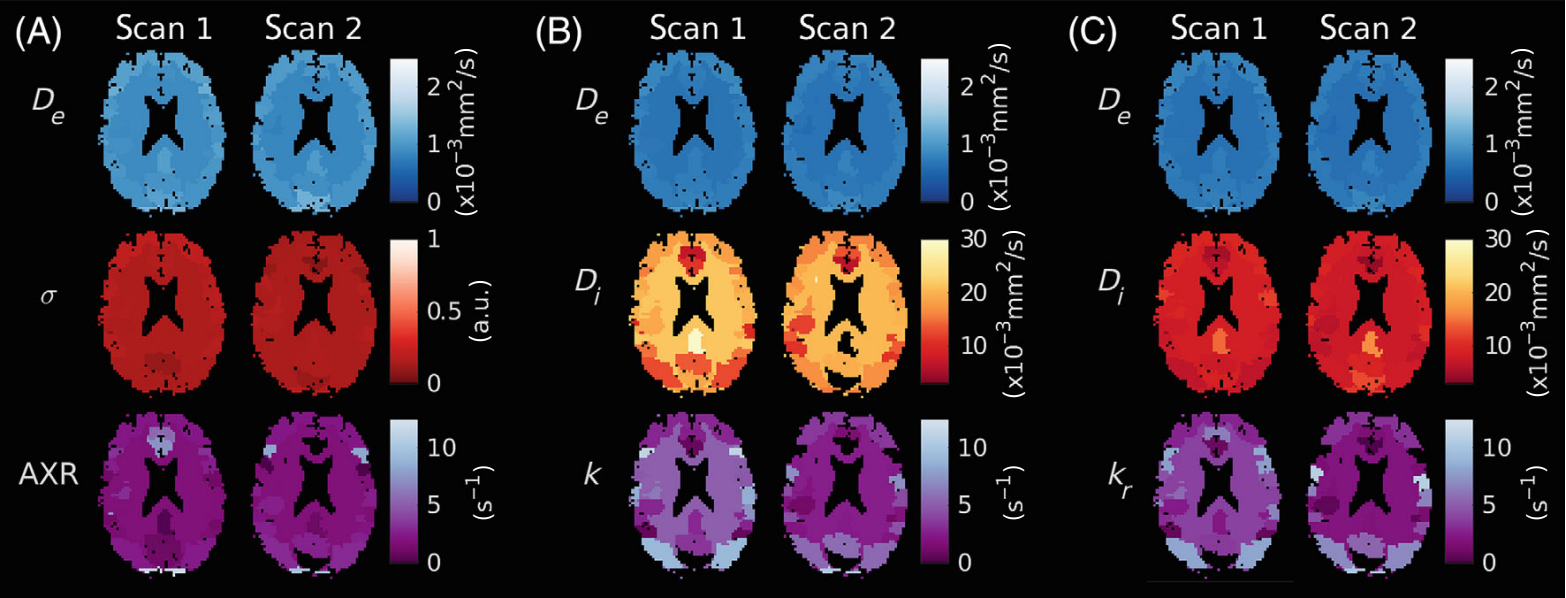
In vivo parameter maps. (A) AXR model. Parameter maps from scans 1 and 2 are shown for the ADC (top row), filter efficiency σ (middle row) and AXR (bottom row). (B) 2CM model. Parameter maps from scans 1 and 2 are shown for $D_{e}$ (top row), $D_{i}$ (middle row) and k (bottom row). (C) $2{\text{CM}}_{r}$ model. Parameter maps from scans 1 and 2 are shown for $D_{e}$ (top row), $D_{i}$ (middle row) and $k_{r}$ (bottom row). All maps display the median value within each ROI; both extreme fit values and masked CSF are shown in black.

Parameter maps from a representative subject are shown in Figure 6; exchange rate maps for all subjects are provided in Figure S7. Good left/right symmetry was observed for all modeling paradigms. Estimates of $D_{i}$ tended toward higher values when derived from the 2CM model compared to the $2{\text{CM}}_{r}$ model, and substantially more noise was observed in the corresponding voxel‐wise fits (shown in Figure S8).

**FIGURE 7:**
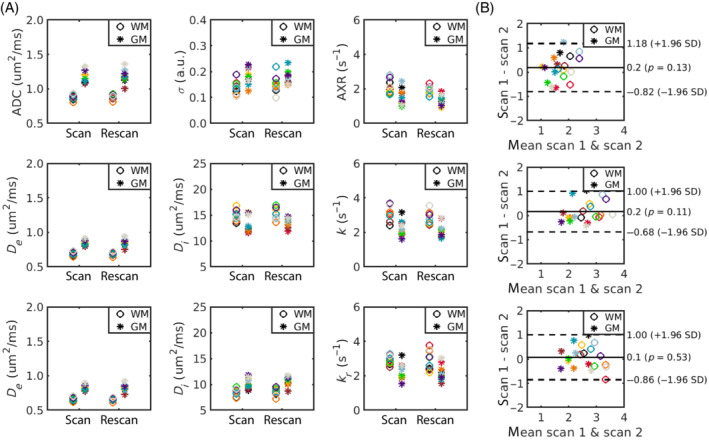
In vivo parameter comparison. (A) Median values across white matter/grey matter (WM/GM) voxels are shown for parameters from the AXR model (top row), 2CM model (middle row) and $2{\text{CM}}_{r}$ model (bottom row). (B) Bland–Altman plots for the exchange rate estimated using the AXR model (top), 2CM model (middle), and $2{\text{CM}}_{r}$ model (bottom). In all plots, each colored marker represents a single subject.

Median WM/GM parameter values are shown in Figure 7A for each subject; Table 3 provides summary statistics over all subjects. Exchange rates in WM and GM were significantly lower when employing the AXR model than when deriving exchange rates using the 2CM and $2{\text{CM}}_{r}$ models (p<0.001 for all comparisons); there were no significant differences in exchange rates between the 2CM and $2{\text{CM}}_{r}$ models (p=0.65/0.82 in WM/GM). Extravascular diffusivity was higher in GM than in WM for all modeling paradigms, as defined by $D_{e}$ in the compartmental models and approximated by the ADC in the AXR model. ADC values were higher than $D_{e}$ for both WM and GM, reflecting the vascular contribution. The intravascular pseudo‐diffusivity $D_{i}$ for both WM and GM was significantly higher in the 2CM model than in the $2{\text{CM}}_{r}$ model (p<0.001 for WM and GM), as observed in simulations (Figure 5).

Bland–Altman plots (Figure 7B) showed negligible bias in exchange rate measurements for all modeling paradigms; however, the 95% limits of agreement were relatively wide. The WM/GM repeatability coefficients were: 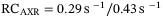, 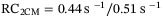 and 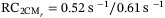. Repeatability coefficients for all other model parameters and the coefficients of variation can be found in Table 3.

The mean SNR in vivo was 66. An example of the acquired data can be found in Figure S5 along with a map of the fitting residuals (Figure S6).

## 5 DISCUSSION

Three modeling paradigms for measuring BBB water exchange using FEXI were implemented and validated using simulations and healthy volunteers. The AXR model previously used for in vivo experiments can be hard to interpret and may not be robust to intercompartmental relaxation time differences, hence the need for a more comprehensive modeling approach; however, parameter estimation from more complex models is invariably more difficult, often resulting in better accuracy but poorer precision in the variables of interest. A more comprehensive compartmental modeling approach for quantifying BBB water exchange was proposed here, enabling for the first time explicit modeling of the blood signal component as well as consideration of relaxation time effects.

Incorporating relaxation time effects during parameter estimation was a key component of this work, as, until now, assuming infinite relaxation times for both compartments has been the convention in applications of FEXI for BBB water exchange measurements.^36^, ^58^ Figure 2A shows that for hypothetical substrates with the same relaxation time in both compartments this assumption can be valid, as exchange rate estimates will be minimally biased. However, for the blood and tissue relaxation times expected in vivo, this assumption introduced errors in both the AXR and 2CM models.

The largest errors due to realistic relaxation times were observed in the AXR model, and arose primarily from intercompartmental $T_{2}$ differences. The greater impact of $T_{2}$ differences (relative to $T_{1}$ differences) can be attributed in part to the combined contribution of the filter and encoding blocks compounding errors and in part to the larger difference between blood and tissue $T_{2}$ values, particularly for WM. Shortening the TE of both filter and encoding blocks was shown in simulations to reduce $T_{2}$‐associated errors (Figure 2B), signifying that MRI systems with enhanced gradient characteristics—which can achieve the same diffusion weighting with a shorter TE^59^—may provide a means of alleviating $T_{2}$‐associated errors in future. The superior accuracy of the 2CM model relative to the AXR model when considering relaxation time effects (Figure 2) was driven by fixing $f_{i}^{\text{eq}}$; as demonstrated in Figure S2, the reduced model stability caused by additionally estimating $f_{i}^{\text{eq}}$ during model fitting generated biases comparable to those from the AXR model.

Fixing parameters, a technique widely discussed in the signal modeling community,^60^, ^61^ can however elicit unintended ramifications. Simulations in this work demonstrated that errors in fixed $f_{i}^{\text{eq}}$ values could indeed induce major biases in estimated exchange rates (Figure 3), although this assumed relatively large errors up to ±50%. In this study of healthy volunteers, where inter‐subject variability in blood volume was not expected, it was considered appropriate to fix $f_{i}^{\text{eq}}$ in the compartmental models. However, in any future studies of clinical disorders that have associated blood volume changes,^7^, ^62^ alternative approaches may be needed. If required, this effect could be negated by providing an independent measure of blood volume. Indeed, an independent measure of blood volume would be beneficial for all modeling paradigms (including the AXR model) in order to convert the average exchange rate derived using this technique into the exchange rate from blood to tissue (often denoted $k_{in}$) to allow for more direct comparisons with other imaging techniques.

The implications of parameter fixing were also considered for the $2{\text{CM}}_{r}$ model, which, in the absence of $T_{1}$ and $T_{2}$ mapping sequences in the MRI protocol of this study, additionally required relaxation times to be fixed. By explicitly modeling finite relaxation times, the $2{\text{CM}}_{r}$ model was less affected by errors in the fixed $f_{i}^{\text{eq}}$, and, perhaps predictably, errors in fixed finite relaxation times demonstrated a comparatively low impact on parameter accuracy compared to assuming infinite relaxation times (Figure 3B). This is an important finding because relaxation times, particularly in blood, are not well defined: literature values for $T_{1,b}$ have been reported between 1.58 and 1.93 s ^49^, ^63^, ^64^ and for $T_{2,b}$ between 0.055 and 0.275 s,^50^, ^63^, ^65^ with differences indicated between males and females, between venous and arterial blood,^64^ and along the vascular tree. However, oxygenation‐dependent $T_{2}$ variations^86^, ^87^ along the vasculature may be largely mitigated in using a mean $T_{2}$ value under the assumption that each large voxel contains a distribution of all vessel types (see Figure S9). Despite these uncertainties, the results here highlight the value in explicitly modeling relaxation times.

In all modeling paradigms, accuracy and precision were lowest for low $f_{i}^{\text{eq}}$ values and fast exchange rates. Fast exchange rates will generally need shorter mixing times than those used in simulations here: for example, at k=20 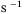 the residence time is τ=1/k=50  ms, meaning that at the simulated mixing times of $t_{m}=200,400$  ms the intravascular component was largely recovered and little discernible difference between the two signals remained. There is also minimal perturbation of the signal by the filter block at very low $f_{i}^{\text{eq}}$, rendering the SNR used in simulations insufficient for accurate quantification of exchange rates. For expected blood volumes in vivo and at exchange rates reflective of the subtle BBB disruption the method is intended to target, distributions of fitted exchange rates (Figure 5) demonstrated that good accuracy and reasonable precision can be expected at clinically feasible SNR levels.

Distributions of $D_{i}$ estimates from the 2CM model (Figure 5) revealed poor accuracy and precision, notably worse than from the $2{\text{CM}}_{r}$ model. One interpretation is that $D_{i}$ captured the majority of biases arising from the infinite relaxation time assumption in the 2CM model, thus also explaining the relative lack of bias in k estimates. Post hoc analysis of the dependence of $D_{i}$ estimates on relaxation times supported this theory (see Figure S4), further highlighting the value in modeling relaxation times in the $2{\text{CM}}_{r}$ model. Corresponding behaviour was observed in the in vivo data (Figure 6), and, while $D_{i}$ estimates from both models were in line with previously reported values between 2 and 15 ${\text{ µm}}^{2}\text{/ms}$,^66^, ^67^, ^68^ the improved visual clarity and lower noise observed in voxel‐wise $D_{i}$ maps from the $2{\text{CM}}_{r}$ model (Figure S8) offered confidence that the lower values generated by this model were also more accurate. This finding may have implications beyond the current study, such as in intravoxel incoherent motion (IVIM) experiments where the assumption of infinite relaxation times in blood and tissue may similarly influence results.

Exchange rate estimates in vivo derived from each modeling paradigm also reflected the behaviors observed in simulations: there was good consistency between k and $k_{r}$, while the AXR was significantly lower (Figure 7 and Table 3). While it could be speculated that relaxation time effects caused the discrepancy between the AXR and k,$k_{r}$ values (as in simulations), the in vivo condition is invariably more complex to interpret, and other factors such as additional exchanging compartments cannot be ruled out for contributing to this finding. The “true” BBB water exchange rate is also unclear: previous studies have reported values for $k_{ie}$ in GM in the range 0.63−4.71 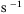.^24^, ^25^, ^26^, ^27^, ^30^, ^36^, ^69^ Possibly the closest comparison may be made with the exchange rates reported by Bai et al.,^36^ who used the FEXI approach to determine the BBB AXR. They found average values across seven subjects of AXR=3.35 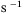 in WM and AXR=4.71 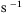 in GM. While the findings are comparable, Bai et al.^36^ report higher AXR values in GM than WM where the opposite was observed in this study (for all modeling paradigms). To date, there is considerable inconsistency in the literature, with some studies supporting the findings here^24^, ^25^, ^69^ and others reporting trends similar to Bai et al.^26^, ^27^, ^36^ Efforts to resolve all of these uncertainties in the field are urgently required if measurements of water exchange are to be considered as reliable biomarkers of BBB function.

All modeling paradigms showed good repeatability and negligible bias in exchange rate measurements between scans (Figure 7B). The repeatability coefficients reported here suggest that the smallest intra‐subject change that can be interpreted to be a true change at the 95% confidence is approximately ±0.6 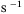 for the compartmental models; this is even lower for the AXR model at ±0.4 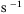, owing to its greater precision. These findings provide confidence that the BBB‐FEXI method could be used to detect subtle damage.

The most appropriate of the three modeling paradigms explored in this work is likely to depend on the context of use. If relaxation time differences or changes are not expected in the chosen study populations then the AXR model may be the prudent choice: exchange rate estimates may be inaccurate, but the bias will be consistent across all subjects and the superior precision (relative to the compartmental models) may enable more subtle changes in exchange rate to be detected. However, there is evidence for relaxation time alterations in many neurological disorders—including dementia,^70^ multiple sclerosis^71^, ^72^, ^73^ and small vessel disease^74^, ^75^, ^76^—as well as in normal aging;^77^, ^78^, ^79^ in these cases, simultaneous alterations in relaxation times and exchange rates may lead to unexpected results if using the AXR model. The $2{\text{CM}}_{r}$ model may then be the more robust choice, although mapping of tissue $T_{1,e}$ and $T_{2,e}$ times on an individual level may be necessary instead of relying on the literature values. Blood relaxation times taken from the literature may still be a reliable choice though, as changes are less likely unless hematocrit levels are altered as, for example, in sickle cell disease.^80^ Alternatively, if blood pseudo‐diffusivity is not a critical parameter for the study, then the 2CM model may also be appropriate and would bypass the requirement for relaxation time mapping, assuming that relaxation time biases continue to influence only $D_{i}$ and not k; however, this warrants further validation. Overall, we anticipate that all BBB‐FEXI modeling paradigms will be well suited for detecting subtle changes during early disease stages, thus providing critical information on pathogenesis. Moreover, the acquisition can be conducted in a clinically feasible time: although single‐slice data were acquired here, it is possible to achieve whole‐brain coverage in a comparable time owing to the long repetition time.

A limitation of the compartmental models is the need to fix $f_{i}^{\text{eq}}$; as this is a parameter liable to change in pathology, the ability to map it would be desirable. Moreover, owing to the low image resolution of the current protocol, it is possible that partial volume effects between WM and GM may introduce biases owing to incorrect assumptions regarding $f_{i}^{\text{eq}}$; however simulations (not shown) indicate that biases under 15% are expected in voxel‐wise estimates, with propagation into the regional parameter estimates subsequently low. Sequence optimisation in future work could improve precision and reduce degeneracy in the model fit, subsequently enabling $f_{i}^{\text{eq}}$ to be left as a free parameter during fitting. Alternatively, an independent measurement of blood volume—such as vascular space occupancy^81^—could be introduced into the imaging protocol to provide this information. The definition of blood volume itself—as the sum of arterial, venous and capillary contributions—is a limitation of all modeling paradigms: because nonpermeable arteries do not contribute to the exchange‐weighted signal, exchange rates may be biased as the recovered intravascular signal will not include the arterial contribution (although exchanged spins will be present in the veins). Estimated exchange rates will therefore be lower than expected at long mixing times given the actual exchange rate, with underestimations up to 60% possible (see Section S10). This is a limitation of any BBB work utilizing the FEXI method, and is an important consideration when comparing results to the literature estimates using alternative methods.

A central assumption throughout this work was that the chosen sequence parameters rendered the signal sensitive to exchange between two compartments only, taken to be the intra‐ and extravascular compartments; however, the components of the BBB that were classified as intravascular were not specifically defined. While sensitivity to cellular exchange in brain tissues is unlikely for the filter *b*‐value used here,^36^, ^42^ it is possible that exchange between perivascular CSF and interstitial water via aquaporin‐4 (AQP4) located on astrocyte endfeet^82^, ^83^ may contribute to the measured water exchange rates. However, presumably the high density of AQP4 water channels covering endfeet and the large area of astocyte endfeet covering capillaries ensures that these membranes are not rate limiting for healthy brain tissues.^84^ If correct, then it may not matter whether the perivascular and astrocyte structures are considered as part of the intra‐ or extravascular compartment. A possible exception may occur if perivascular water has a substantially different $T_{2}$ to blood or tissue. Furthermore, in pathologies where the endothelial tight junctions are damaged, astrocyte endfeet may pose a more significant barrier to water exchange, particularly if AQP4 polarization is altered or AQP4 levels are downregulated.^85^ In this case, these membranes may become rate limiting. Nevertheless, this is a potentially interesting direction for future water exchange research.

## 6 CONCLUSIONS

The impact of relaxation time effects, the repeatability and the clinical feasibility of three biophysical models of BBB water exchange applied to FEXI‐style acquisitions were evaluated. Relaxation time effects—which are intrinsically entwined with exchange effects—can introduce substantial biases into exchange rate estimates; this was particularly evident in the AXR model. The two‐compartment models, which are a step toward more comprehensive modeling of BBB exchange mechanisms, were more robust to relaxation time biases. The healthy volunteer repeatability of BBB exchange rate estimates, evaluated here for the first time, demonstrates that the BBB‐FEXI technique offers a reliable approach for detecting subtle changes in BBB integrity clinically.

## Supporting information




**Table S1.** Model assumptions
**Figure S2.** Assumption of infinite relaxation times with $f_{i}^{\text{eq}}$ an additional free parameter in the 2CM model
**Figure S3.** Parameter distributions (variable SNR)
**Figure S4.** Dependence of *D*_*i*_ on relaxation times in the 2CM model
**Figure S5.** In vivo data
**Figure S6.** In vivo residuals
**Figure S7.** In vivo exchange rate maps (all subjects; regional fits)
**Figure S8.** In vivo parameter maps (single subject; voxel‐wise fits)
**Figure S9.** Dependence of *k*_*r*_ on blood *T*_2_ and oxygenation level
**Section S10.** Definition of blood volume

## APPENDIX A EIGENVALUES AND EIGENVECTORS OF EXCHANGE‐RELAXATION MATRIX EXPONENTIAL

### A.1

The eigenvalues λ and eigenvectors v of the matrix $R_{1}+k$ are given by:

(A1)
$$\lambda_{\pm }=\frac{B\pm \sqrt{A}}{2}\;;\;v_{\pm }=\frac{2\left(R_{1,e}+k_{ei}\right)-\left(B\mp \sqrt{A}\right)}{2k_{ie}},$$

where

(A2)
$$\begin{array}{ll} A={\left(R_{1,e}-R_{1,i}+k_{ei}-k_{ie}\right)}^{2}+4k_{ie}k_{ei} & \end{array}$$

(A3)
$$\begin{array}{ll} B=R_{1,e}+R_{1,i}+k_{ie}+k_{ei}. & \end{array}$$

Elements of the matrix exponential $C=e^{-\left(R_{1}+K\right)t}$, computed as $C=Ve^{\Lambda }V$ with V a matrix of eigenvectors and Λ a diagonal matrix of eigenvalues, are thus:

(A4)
$$\begin{array}{ll} C_{1,1} & =\frac{1}{2\sqrt{A}}\left[\left(R_{1,i}-R_{1,e}+\sqrt{A}-k_{ei}+k_{ie}\right)e^{-\frac{t_{m}}{2}\left(B+\sqrt{A}\right)}\right.\\ & \;\left.-\left(R_{1,i}-R_{1,e}-\sqrt{A}-k_{ei}+k_{ie}\right)e^{-\frac{t_{m}}{2}\left(B-\sqrt{A}\right)}\right], \end{array}$$

(A5)
$$\begin{array}{ll} C_{1,2} & =-\frac{k_{ei}}{\sqrt{A}}\left[e^{-\frac{t_{m}}{2}\left(B+\sqrt{A}\right)}-e^{-\frac{t_{m}}{2}\left(B-\sqrt{A}\right)}\right]. \end{array}$$

(A6)
$$C_{2,1}=-\frac{k_{ie}}{\sqrt{A}}\left[e^{-\frac{t_{m}}{2}\left(B+\sqrt{A}\right)}-e^{-\frac{t_{m}}{2}\left(B-\sqrt{A}\right)}\right].$$

(A7)
$$\begin{array}{ll} C_{2,2} & =\frac{1}{2\sqrt{A}}\left[\left(R_{1,i}-R_{1,e}+\sqrt{A}-k_{ei}+k_{ie}\right)e^{-\frac{t_{m}}{2}\left(B-\sqrt{A}\right)}\right.\\ & \;-\left.\left(R_{1,i}-R_{1,e}-\sqrt{A}-k_{ei}+k_{ie}\right)e^{-\frac{t_{m}}{2}\left(B+\sqrt{A}\right)}\right]. \end{array}$$
