# Supplementary material for: Blood‐brain barrier water exchange measurements using FEXI: Impact of modeling paradigm and relaxation time effects
Source: Magn Reson Med. 2023 Mar 9;90(1):34–50. doi: 10.1002/mrm.29616 (PMC10962589; doi:10.1002/mrm.29616)
Supplement: Supplementary file 1 — Table S1. Model assumptions Figure S2. Assumption of infinite relaxation times with fieq an additional free parameter in the 2CM model Figure S3. Parameter distributions (variable SNR) Figure S4. Dependence of D i on relaxation times in the 2CM model Figure S5. In vivo data Figure S6. In vivo residuals Figure S7. In vivo exchange rate maps (all subjects; regional fits) Figure S8. In vivo parameter maps (single subject; voxel‐wise fits) Figure S9. Dependence of k r on blood T 2 and oxygenation level Section S10. Definition of blood volume [file MRM-90-34-s001.pdf]

729 # Supporting Figures and Tables

**Table S1. Model assumptions.**

The free and fixed parameters in each modelling paradigm are specified, along with assumptions about relaxation times and the number of exchanging compartments. All models additionally assume that compartments are well-mixed.

| | AXR | 2CM | 2CM$_r$ |
|---|---|---|---|
| Free parameters | ADC, $\sigma$, AXR | $D_i$, $D_e$, $k$ | $D_i$, $D_e$, $k_r$ |
| Fixed parameters | - | $f_i^{eq}$ | $f_i^{eq}$, $T_{1,e}$, $T_{1,i}$, $T_{2,e}$, $T_{2,i}$ |
| Relaxation times | $\infty$ | $\infty$ | finite |
| No. compartments | non-specific | 2 | 2 |

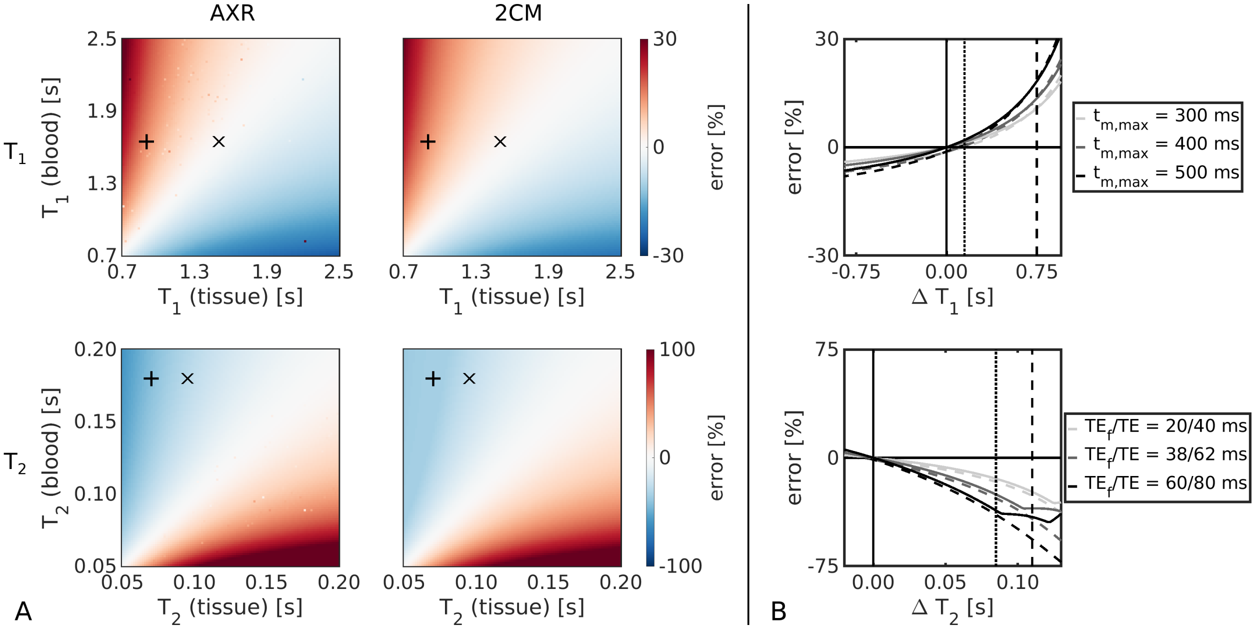


**Figure S2. Assumption of infinite relaxation times with $f_i^{eq}$ an additional free parameter in the 2CM model.**

**(A)**. The errors in exchange rate estimates are shown for a range of finite compartmental $T_1$ (top row) and $T_2$ (bottom row) values for the AXR (left column) and 2CM (right column) models. Expected blood/tissue values in WM (+) and GM (×) are highlighted. **(B)**. The impact of different maximum mixing times on the error in exchange rate estimates is shown for a range of $T_1$ differences (top); the impact of different echo times is shown for a range of $T_2$ differences (bottom). The AXR and 2CM models are represented by the dashed and solid lines respectively. Expected relaxation time differences ($\Delta T = T_i - T_e$) in WM and GM in vivo are indicated by the vertical dashed and dotted lines respectively. With $f_i^{eq}$ an additional free parameter in the 2CM model, biases were comparable to those in the AXR model. Note that the AXR results are unchanged from Figure 2, but are reproduced here for ease of comparison.

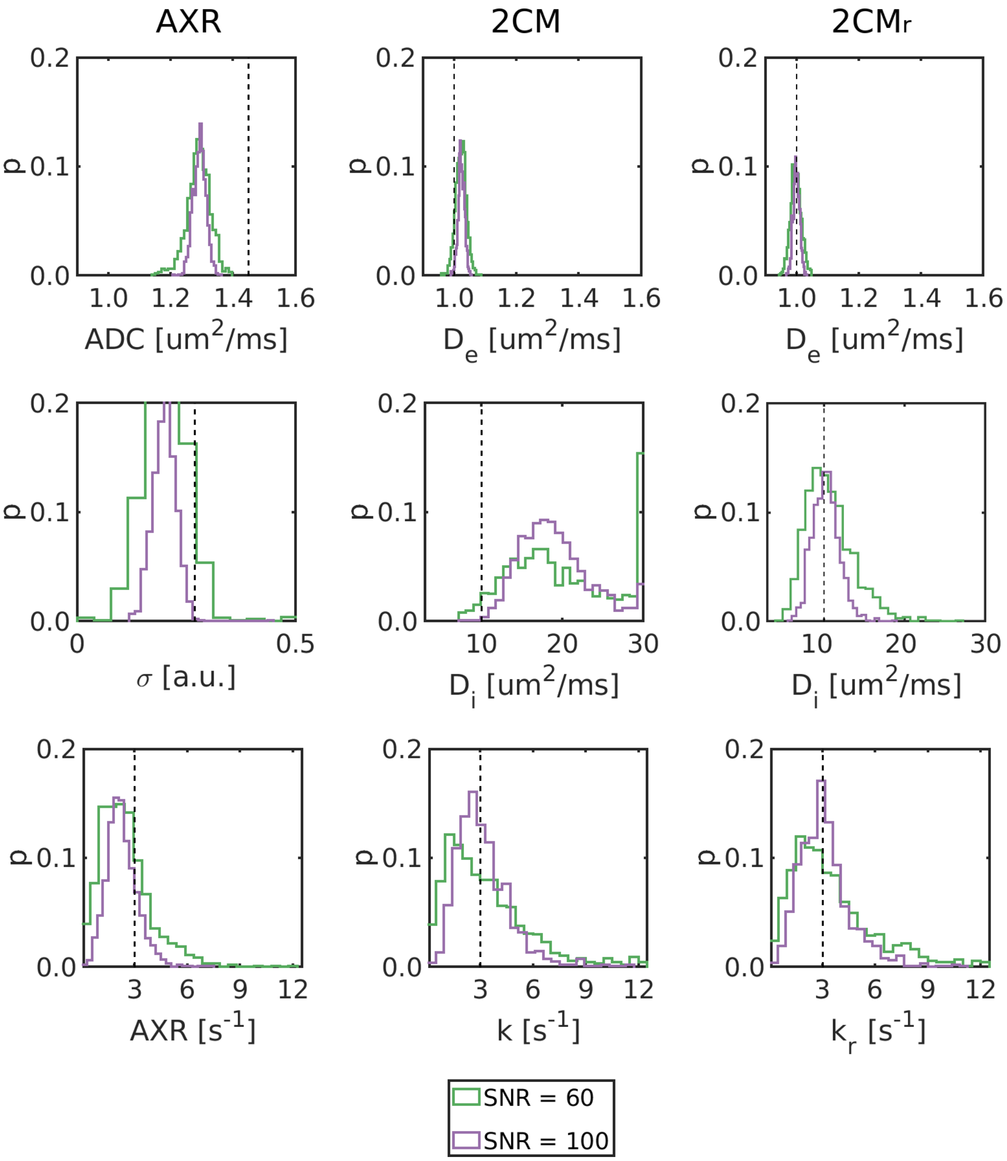


**Figure S3. Parameter distributions (variable SNR).**

Model parameter probability distributions are shown for the AXR model (left column), 2CM model (centre column) and $2CM_r$ model (right column) for one set of generative parameter values ($f_i^{eq} = 0.05$; $k = 3\,s^{-1}$) at SNR $= 60, 100$. Ground truth (generative) values are represented by the dashed lines. Biases were evident in all parameters of the AXR model, with the AXR and ADC notably underestimated. The 2CM model also showed an underestimation in $k$ at SNR $= 60$; however, this was no longer evident at SNR $= 100$.

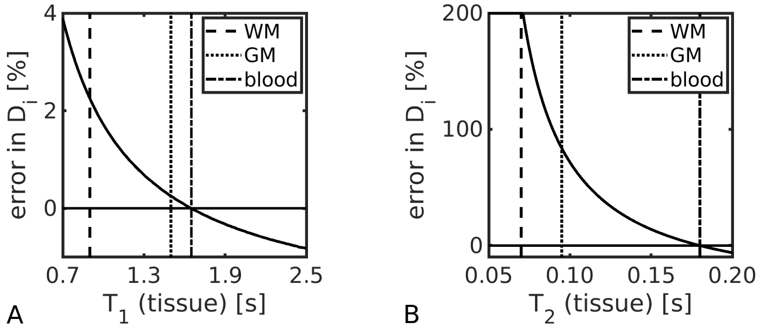


**Figure S4. Dependence of $D_i$ on relaxation times in the 2CM model.**

**(A)**. Longitudinal relaxation time effects. Signals were generated using the 2CM$_r$ model with: $T_{1,e} = 0.7 - 2.5\,\mathrm{s}$, $T_{1,i} = 1.65\,\mathrm{s}$, $T_{2,e} = T_{2,i} = \infty$, $f_i^{eq} = 0.05$, $k = 3\,\mathrm{s}^{-1}$. **(B)**. Transverse relaxation time effects. Signals were generated using the 2CM$_r$ model with: $T_{1,e} = T_{1,i} = \infty$, $T_{2,e} = 0.05 - 0.20\,\mathrm{s}$, $T_{2,i} = 0.18$, $f_i^{eq} = 0.05$, $k = 3\,\mathrm{s}^{-1}$. In both plots, signals were fitted using the 2CM model (i.e. assuming in (A) that $T_{1,e} = \infty$ and in (B) that $T_{2,e} = \infty$). As the simulated tissue relaxation times approached the blood relaxation times (vertical dot-dashed lines), errors in the intravascular diffusivity tended to 0. This correlation between underlying relaxation time and error in $D_i$ suggests that the majority of biases arising from the infinite relaxation time assumption in the 2CM model are captured by the $D_i$ estimates, leaving a relative lack of bias in $k$ estimates.

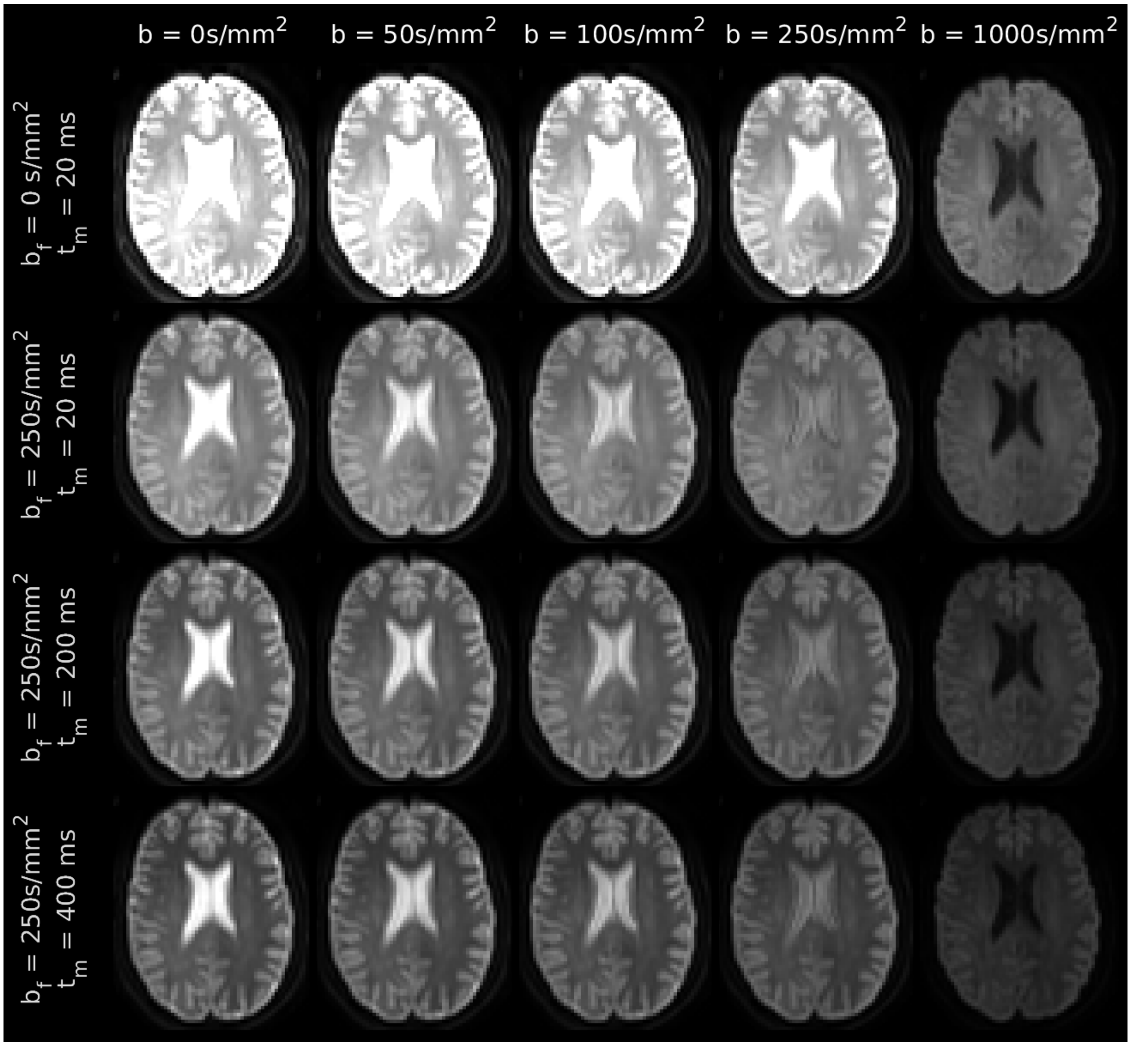


**Figure S5. In vivo data.**

Example images from the four BBB-FEXI acquisitions (rows) at each encoding *b*-value (columns) are shown for a representative subject. All images are shown with the same intensity scaling.

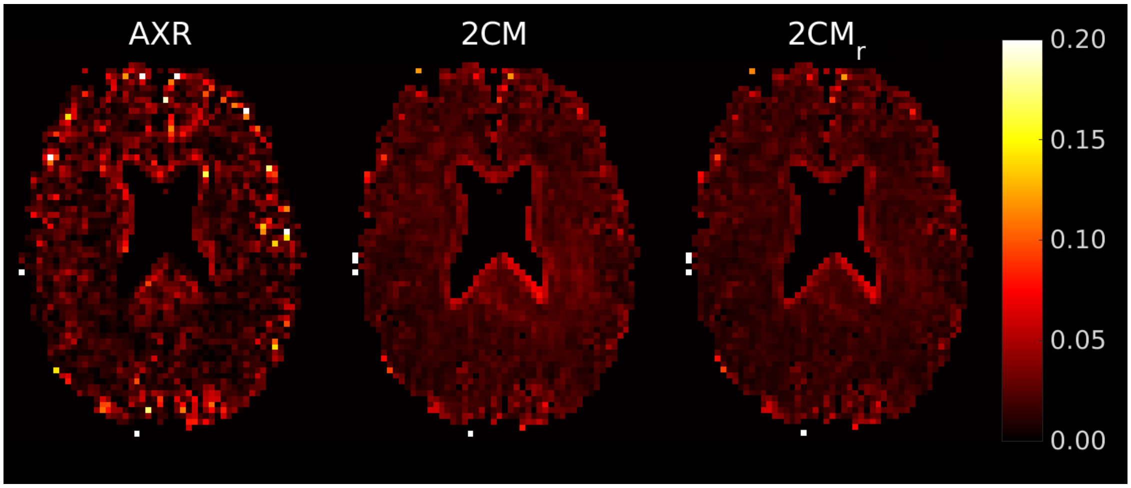


**Figure S6. In vivo residuals.**

Maps of the normalised root mean square error (NRMSE) from the three modelling paradigms are shown for a representative subject. The NRMSE in each voxel was computed as NRMSE $= \sqrt{\sum_{i=1}^{N}\left(\hat{S}_i - S_i\right)^2} / \sqrt{\sum_{i=1}^{N}\left(\bar{S} - S_i\right)^2}$, where $S_i$ is the measured signal, $\hat{S}_i$ is the model-predicted value, $\bar{S}$ is the mean of the measured values and $N$ is the number of acquisitions. All models fitted the data comparably.

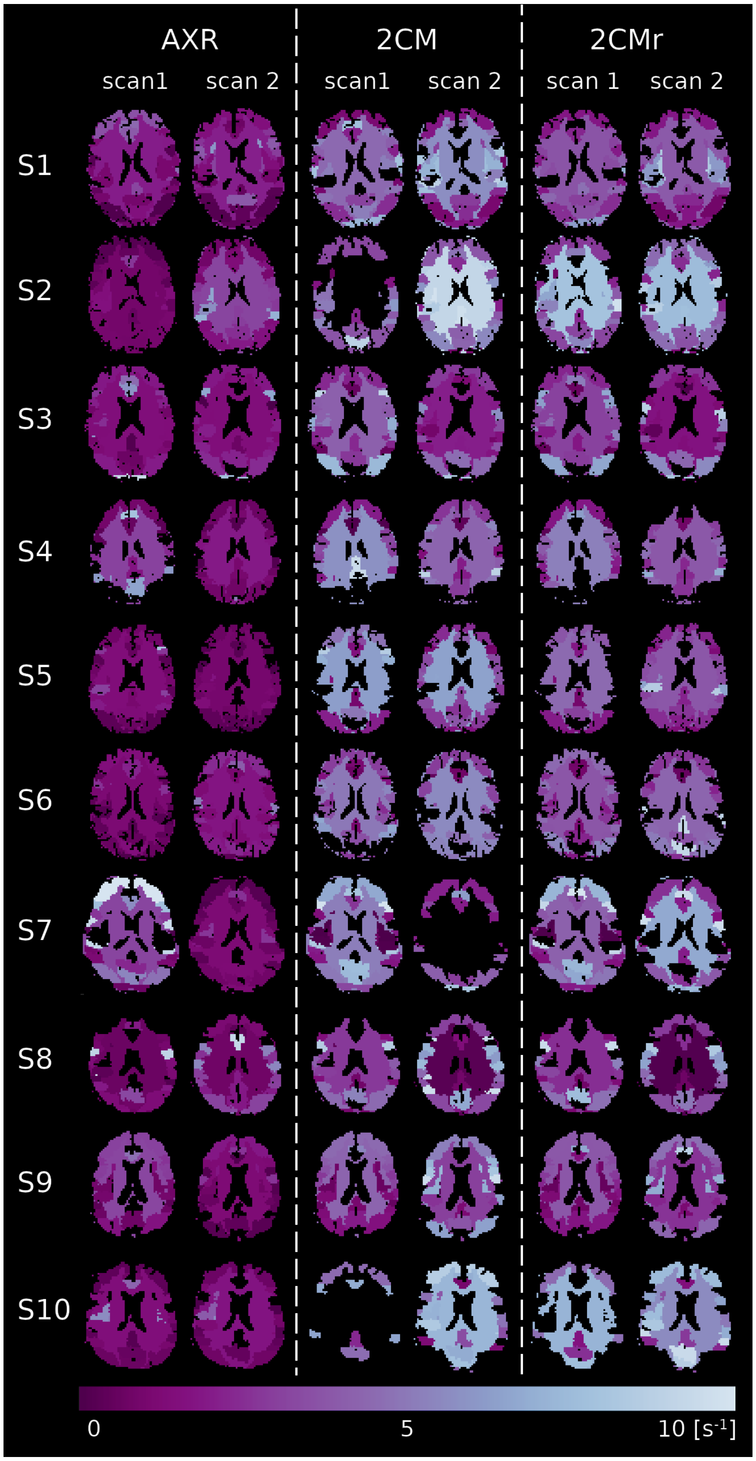


**Figure S7. In vivo exchange rate maps (all subjects; regional fits).**

Exchange rate maps derived from the AXR model (left), 2CM model (centre) and $2CM_r$ model (right) are shown for each subject (rows). Displayed exchange rates are the median within each ROI; black voxels indicate extreme values (exchange rate $\geq 12.5\,s^{-1}$) and masked CSF.

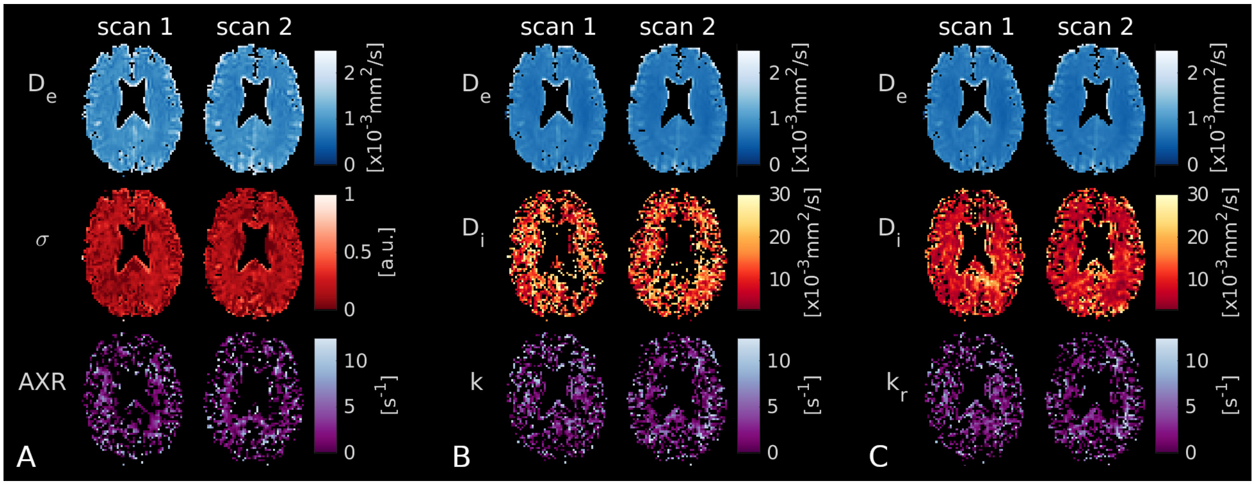


**Figure S8. In vivo parameter maps (single subject; voxel-wise fits).**

**(A)**. AXR model. Parameter maps from scans 1 and 2 are shown for the ADC (top row), filter efficiency $\sigma$ (middle row) and AXR (bottom row). **(B)**. 2CM model. Parameter maps from scans 1 and 2 are shown for $D_e$ (top row), $D_i$ (middle row) and $k$ (bottom row). **(C)**. 2CM$_r$ model. Parameter maps from scans 1 and 2 are shown for $D_e$ (top row), $D_i$ (middle row) and $k_r$ (bottom row). In all maps, both extreme fit values and masked CSF are shown in black.

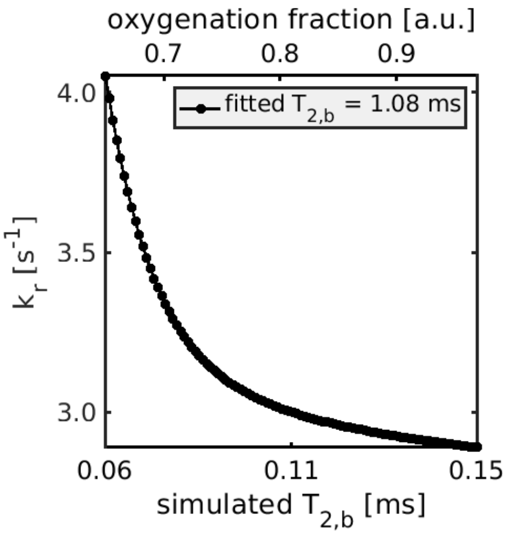


**Figure S9. Dependence of $k_r$ on blood $T_2$ and oxygenation level.**

Signals were generated using the 2CM$_r$ model with: $T_{1,e} = T_{1,i} = \infty$, $T_{2,e} = 0.095\,\text{s}$, $f_i^{eq} = 0.05$, $k = 3\,\text{s}^{-1}$ and 100 $T_{2,i}$ values uniformly distributed between $0.062\,\text{s} \leq T_{2,i} \leq 0.154\,\text{s}$, corresponding to oxygenation levels between $65 - 100\,\%$ (86; 87). The 2CM$_r$ model was fitted back to each of the 100 the synthetic signals using a mean $|T_{2,b}| = 0.108\,\text{s}$; the exchange rate estimated at each underlying $T_{2,i}$ is plotted here. The average of $k_r$ estimates over the range of $T_{2,b}$ values was equivalent to the $k_r$ estimated using the average $T_{2,b}$ and equal to the ground truth $k$, i.e. $\left|k_r\left(T_{2,b}\right)\right| = 3.1\,\text{s}^{-1}$ and $k_r\left(\left|T_{2,b}\right|\right) = 3.0\,\text{s}^{-1}$, indicating that the use of a mean $T_{2,b}$ for the capillaries was an appropriate approach.

# Section S10. Definition of blood volume

Blood volume is defined as the sum of arterial, venous and capillary contributions; however, because non-permeable arteries do not contribute to the exchange-weighted signal, exchange rates may be biased as the recovered intravascular signal will not include the arterial contribution (although exchanged spins will be present in the veins) and will therefore be lower than expected at long mixing times given the actual exchange rate. This is a limitation of any BBB work utilising the FEXI method. The degree of underestimation was approximated here using the 2CM$_r$ model with infinite relaxation times; this formulation allowed the exchange rate to be modulated by different exchanging fractions while removing any confounding relaxation effects, meaning that a quantitative estimation of errors arising solely from the blood volume definition could be elucidated.

The total intravascular volume fraction can be defined as $f_i^{eq} = f_a^{eq} + f_v^{eq} + f_c^{eq}$, where the subscripts $a$, $v$ and $c$ denote the arterial, venous and capillary contributions respectively. It was assumed that the arterial and venous components each contributed 20 % of the total blood volume (i.e. $f_a^{eq} = f_v^{eq} = 0.2 \times f_i^{eq}$). Ground truth signals were simulated such that the filter block acted on the total blood fraction but exchange during the mixing time occurred only with the capillary fraction (i.e. the total recovered intravascular signal fraction $f_i\,(t_m)$ was limited by $f_c^{eq}$). Signals were generated for different combinations of mixing times (Table S10). The AXR and 2CM models were fitted back to the simulated data and the resulting bias in exchange rate calculated as a function of maximum mixing time (Figure S10). It can be seen that for the acquisition used in this work (i.e. with maximum $t_m = 400\,\text{ms}$) the exchange rate may be underestimated by up to 60 %.

**Table S10. Simulated acquisition parameters.**

| | BBB-FEXI | | | |
|---|---|---|---|---|
| $b$ [s/mm$^2$] | 0, 50, 100, 250, 1000 | | | |
| $b_f$ [s/mm$^2$] | 0 | 250 | 250 | 250 |
| $t_m$ [ms] | | | | |
| (i) | 20 | 20 | 50 | 100 |
| (ii) | 20 | 20 | 100 | 200 |
| (iii) | 20 | 20 | 200 | 400 |
| (iv) | 20 | 20 | 300 | 600 |

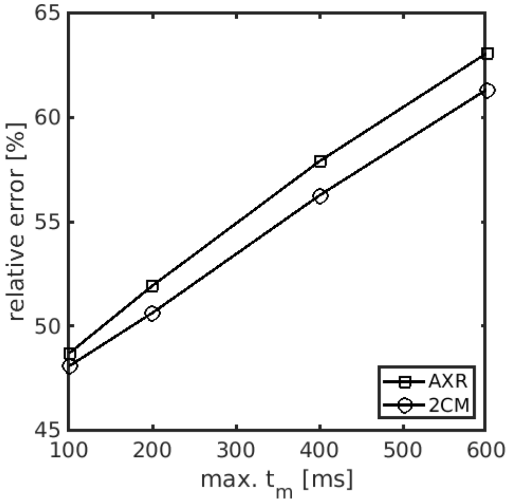


**Figure S10. Relative error in exchange rate.**

The bias arising from an exchanging blood volume fraction smaller than the total suppressed blood fraction is shown for the AXR and 2CM models.
